# Malaria Vectors and *Plasmodium* Transmission in Malaria-Endemic Localities of Colombia

**DOI:** 10.3390/tropicalmed9110260

**Published:** 2024-11-01

**Authors:** Stefani Piedrahita, Margarita M. Correa

**Affiliations:** Grupo Microbiología Molecular, Escuela de Microbiología, Universidad de Antioquia, Calle 70 No. 52-21, Medellín 050010, Colombia; estefani.piedrahita@udea.edu.co

**Keywords:** malaria, transmission, *Plasmodium*, *Anopheles*, Colombia

## Abstract

*Anopheles* species composition, abundance, and *Plasmodium* natural infection are important aspects to assess malaria transmission risk. In this study, these aspects were evaluated in a large number of localities in the most important malaria-endemic regions of Colombia. Mosquitoes were collected from 2012 to 2015 in 55 localities of northwestern and western Colombia. *Anopheles* species composition, abundance, and *Plasmodium* infection were estimated. A total of 13,218 *Anopheles* specimens were evaluated. The highest species richness was detected in the northwest, where the main vectors, *An. albimanus* (27.8%) and *An. nuneztovari* (26.7%), were the most abundant species. In the west, *An. nuneztovari* predominated (51.6%), followed by *An. darlingi* (29.2%). Six species were infected with *Plasmodium, An. darlingi*, *An. nuneztovari*, *An. albimanus*, *An. calderoni*, *An. triannulatus*, and *An. braziliensis*. Results showed that in these localities the main Colombian vectors are widely spread, which suggests a high malaria risk. Notably, this study is the first to report *An. braziliensis* from Colombia as being naturally infected with *Plasmodium*. Infection results for species that are suspected local vectors indicate the importance of conducting further studies to assess their epidemiological importance. This information provides the basis for the application of directed vector control strategies that are species-specific.

## 1. Introduction

*Anopheles* vectors transmit *Plasmodium*, the parasite causing human malaria. Parasite transmission occurs when an infected *Anopheles* vector inoculates sporozoites into the human blood during a bite [1,2,3]. In Colombia, *Plasmodium vivax* Grassi and Feletti, 1890, and *Plasmodium falciparum* Welch, 1897, are the predominant species [4]. In the year 2023, a total of 102.457 cases were reported, indicating an increase in malaria incidence with respect to 2022 [5]. Historically, the most malaria-endemic regions are located in northwestern and western Colombia [6]. These areas present environmental conditions that favor the development and presence of the Colombian main vectors, *Anopheles nuneztovari* Gabaldón, 1940, *Anopheles darlingi* Root, 1926, and *Anopheles albimanus* Wiedemman, 1920, and various species that are potential local vectors [7,8,9].

Previous studies carried out in endemic localities of Colombia have recorded the *Anopheles* species naturally infected with *Plasmodium*. In northwestern Colombia, *An. darlingi* [10,11] and *An. nuneztovari* were detected infected with *P. vivax* [12]. Meanwhile, in western Colombia, in the Pacific region, *An. albimanus* was detected with *P. vivax* and *P. falciparum* [13], and *An. nuneztovari* with *P. vivax* [14,15,16,17]. Regarding the local vectors, *Anopheles neivai* Howard, Dyar, and Knab, 1912 [13], and *Anopheles calderoni* Wilkerson, 1991, were reported to be infected with *Plasmodium* sp. [11,18]. Furthermore, species such as *An. triannulatus*, a local vector in Brazil [19], was detected in northwestern Colombia to be infected with *Plasmodium* sp. in abdomen DNA samples [17,20]; therefore, its implication as a vector is not conclusive. To assess malaria risk, it is important to identify the species responsible for transmission determining their natural infection; in addition, species abundance is a factor that may increase human exposure to infected vectors. Determination of these factors is crucial to direct malaria prevention measures. Therefore, this study was carried out in a large number of localities of the most malaria-endemic regions of northwestern and western Colombia. The aim was to provide data on *Anopheles* composition, abundance, and natural infection with *Plasmodium*. This information provides the basis for the application of targeted vector control measures.

## 2. Materials and Methods

### 2.1. Mosquito Sampling and Processing

**Figure 1:**
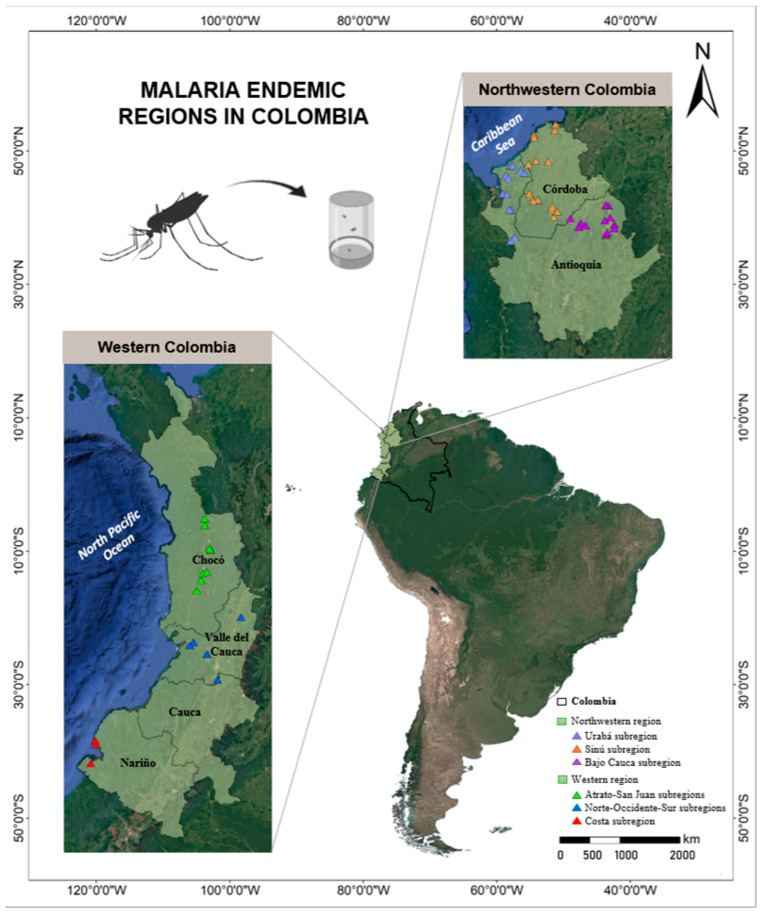
Mosquito collection site in the field. The mosquitoes were collected in the most malaria-endemic regions of northwestern and western Colombia.

The analyzed *Anopheles* mosquitoes were collected in a previous study, carried out to evaluate ecological niche models for the Colombian main vector species [9,21]. In the present work, the specimens were evaluated for their natural infection, considering species abundance and composition. Briefly, *Anopheles* mosquitoes in 55 localities corresponding to 30 municipalities were selected: 20 in northwestern and 10 in western Colombia (Figure 1). The criteria for sampling these localities included a high incidence of malaria cases and accessibility, which depended on situations of social unrest. Adult mosquitoes were collected within the period of December 2012 to November 2015, using Human Landing Catch (HLC); indoor and outdoor (~10 m from the house) collections were performed, between 18:00 and 00:00 h, for three nights in each locality. In addition, some mosquitoes were collected resting on animals, corrals, vegetation, and house walls. Mosquito collection details, processing, and identification were as previously described [15]. Regarding the HLC, informed consent and protocol were approved by the Bioethics Committee, Facultad Nacional de Salud Pública of Universidad de Antioquia (Act 063; 07-41-082). All the specimens of problematic species, e.g., species complexes and sister species with overlapping morphology, as well as a percentage (10–20%) of non-problematic species, were confirmed by PCR-RFLP-ITS2 or *COI* barcode [9,22] using DNA extracted from the mosquito abdomen. In the case of a few specimens, such as *An. squamifemur*, only one specimen was available; it was only identified by morphological characters because it was included in the biological collection of the group. Furthermore, for specimens of *An. apicimacula*, *An. calderoni*, *An. malefactor*, *An. neomaculipalpus* and near-*An. peryassui*, each species was assigned by ITS2 and *COI* barcode [9].

### 2.2. Detection of Plasmodium Natural Infection

Natural infection in *Anopheles* mosquitoes was evaluated as follows: First, two independent Enzyme-Linked Immunosorbent Assays (ELISAs) using monoclonal antibodies directed to the circumsporozoite Protein-*CSP* specific for *P. falciparum* and *P. vivax* VK247/VK210 were applied to mosquitoes processed in pools of up to five thoraxes and heads of the same species and locality, following standard procedures [13,23]. ELISA-positive pools were confirmed by a second ELISA. *Plasmodium* detection was also performed by nested PCR, using genus and species-specific primers targeting the small ribosome subunit [12,24] in DNA extracted from mosquito abdomens [25]. These were processed in pools of up to five *Anopheles* abdomens of the same species. Individual mosquitoes of a positive DNA pool were confirmed using species-specific primers for *P. vivax*, *P. falciparum*, and *P. malariae* [24]. Furthermore, a conventional PCR that amplifies a 500 bp region of the *Plasmodium* Cytochrome Oxidase III—*COXIII* gene was optimized in the laboratory to increase the probability of detecting infected mosquitoes [16,26].

### 2.3. Abundance, Diversity and Infection Index

Abundances were estimated for each *Anopheles* species, by locality and region; and were expressed in absolute and relative values. Species diversity was evaluated by locality using the Shannon (H) index [27]. Infection rate (IR) was expressed as the number of *Plasmodium* positive specimens of each species and locality (np), over the total number of mosquitoes analyzed (tn), expressed as a percentage [IR = (np/tn) × 100] [10]. In addition, a confidence interval (CI 95%) was calculated to indicate the reliability of the estimated value under the assumption of a binomial distribution, using EPIDAT v. 3.1 [28].

## 3. Results

### 3.1. Composition, Abundance, and Species Diversity in Northwestern Colombia

**Figure 2:**
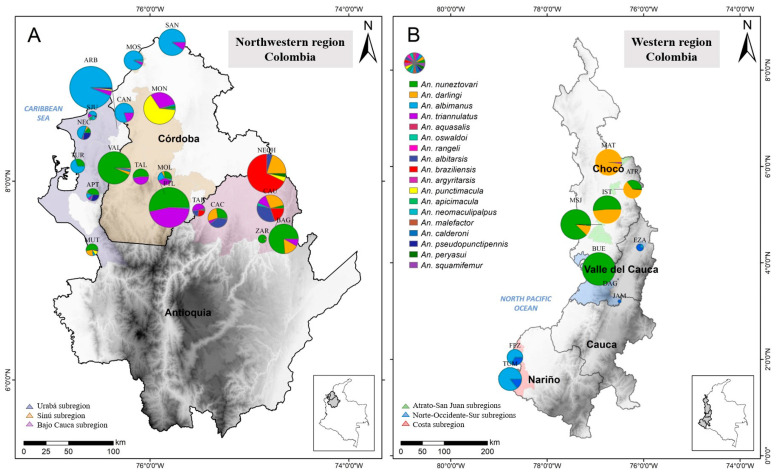
Composition and abundance of *Anopheles* mosquitoes collected in the most malaria-endemic region of Colombia. (**A**) Northwestern region. (**B**) Western region. ZAR: Zaragoza; BAG: El Bagre; NECH: Nechí; CAU: Caucasia; CAC: Cáceres; TAR: Tarazá; ARB: Arboletes; NEC: Necoclí; MUT: Mutatá; APT: Apartadó; TUR: Turbo; SJU: San Juan de Urabá; MON: Montería; VAL: Valencia; MOS: Moñitos; SAN: San Antero; MOL: Montelíbano; PTL: Puerto Libertador; CAN: Canalete; TAL: Tierralta. Acronyms of three-letters correspond to the names of the municipalities sampled.

A total of 13,218 *Anopheles* mosquitoes were evaluated; 9871 mosquitoes were collected from localities in northwestern Colombia, 73.4% by HLC, 26.3% by animal bait, and the remaining 0.3% were resting outdoors. From the 3347 specimens from western Colombian localities, 99.5% were collected by HLC, and the remaining 0.5% were by animal bait and resting outdoors. In the northwest, 18 species were identified, belonging to three subgenera: *Nyssrhynchus* (*n:* 10), *Anopheles* (*n:* 7), and *Lophopodomyia* (*n:* 1); the latter specimen corresponded to *An. squamifemur* based on morphological identification, but this was not molecularly confirmed (Table 1). *Anopheles albimanus* (27.8%) was the most abundant species, mostly because of its dominance in some coastal localities of the Urabá and Sinú subregions (Figure 2). Following this, *An*. *nuneztovari* represented 26.7% of the specimens, mostly because its presence in localities of the Bajo Cauca and Sinú subregions; this species was found in most localities except for three coastal localities. Other species were found in lower abundances, e.g., *An*. *triannulatus* (14.7%), *An*. *braziliensis* (10.1%), *An*. *darlingi* (7.2%), *An*. *punctimacula* (6.4%), *An*. *albitarsis* s.l. (4.1%), and *An*. *pseudopunctipennis* (1.2%). Ten species make up the remaining 1.8% of the total (Table 1).

The highest species richness was detected in the Bajo Cauca subregion, with 3081 specimens encompassing 15 species. The most abundant species in Bajo Cauca was *An*. *braziliensis* (32.3%), with one municipality contributing close to 80% of the total *An*. *braziliensis* collected in the region (Table 1 and Figure 2). The second most abundant species was *An. nuneztovari* (23.4%), followed by *An. darlingi* (19.9%); of notice, in localities where one of these vectors was present, the other was in low abundance or not present. The other 10 species were found at low densities, including specimens that for morphology were assigned as *Anopheles* near *peryassui* and *An. squamifemur*. Given the low number of specimens of these species, future surveys involving the collection of additional samples that allow for confirming their identity will help to elucidate if they represent two new records for this geographical area. In the Urabá subregion, a relatively low mosquito density was detected in comparison with the previously mentioned subregions (*n* = 2139). The predominant species was *An*. *albimanus* (75.5%) (Figure 2). Although this species was found in all Urabá municipalities, most of the specimens were collected in the localities of coastal Arboletes municipality (*n* = 1368), which was the locality that also showed the greatest anopheline abundance and the greatest species richness with 9 of the 12 species detected in Urabá (Table 1). Regarding the Sinú subregion, a total of 4651 specimens were collected. The most abundant species in this subregion were *An*. *nuneztovari* (36.7%), *An*. *triannulatus* (25%), *An*. *albimanus* (24.2%), and *An*. *punctimacula* (12%) and other species accounted for 2.1% of the abundance: *An*. *darlingi*, *An*. *aquasalis*, *An*. *oswaldoi* s.l., *An*. *rangeli*, *An*. *albitarsis* s.l., *An*. *pseudopunctipennis*, and *An*. *malefactor* (Table 1).

*Anopheles* mosquito diversity in northwestern Colombia was, in general, low (H < 2). The Bajo Cauca subregion presented the highest diversity with Shannon index values ranging from 0.15 to 1.93 and one locality, Cáceres, contributed the most to this diversity; in the Urabá subregion, diversity ranged from 0.08 to 1.43, mostly attributed to localities of municipality Apartadó. Contrary, the Arboletes localities show low diversity values because of *An. albimanus* dominance. Diversity values for the Sinú subregion were <1 in 12 of 15 localities. The greatest diversity was detected in the Montelíbano localities with values between 1.38 and 1.22.

### 3.2. Composition, Abundance, and Species Diversity in Western Colombia

A total of 3347 *Anopheles* mosquitoes from western Colombia were evaluated. Nine *Anopheles* species belonging to three subgenera were detected: *Nyssrhynchus* (four species), *Anopheles* (four species), and *Kerteszia* (one species) (Table 2). *Anopheles nuneztovari* was the most abundant species (51.6%), predominating in localities of Medio San Juan (Atrato-San Juan subregions) and in Buenaventura (Norte-Occidente-Sur subregions); in the latter, it was the only species collected. *An*. *darlingi* followed (29.2%), only collected in the Atrato-San Juan subregions, predominating in the localities of Medio Atrato, Atrato, and Istmina; this was followed by *An*. *albimanus* (13.7%) and *An*. *calderoni* (4.5%), which predominated in Tumaco and Francisco Pizarro (Costa subregion) (Figure 2). The species *An*. *apicimacula*, *An*. *neivai*, *An*. *triannulatus*, and *An*. *punctimacula* represented 1% (Table 2).

### 3.3. Natural Infection by Plasmodium

From 13,218 mosquitoes tested for *Plasmodium* natural infection, 12 gave a positive result: 6 in each region, northwest and west. All infected specimens were collected by HLC. Two specimens *An. darlingi* were infected with *P. falciparum* (IR: 0.66) and with *P. vivax* (IR: 0.37); three *An*. *nuneztovari* were infected with *P. falciparum* (IR: 0.35) and *Plasmodium* sp. (IR: 0.13–1.67); two *An*. *albimanus* were infected with *P. falciparum* (IR: 0.07) and *Plasmodium* sp. (IR: 0.30); and *An*. *triannulatus* (IR: 0.35–10), *An*. *braziliensis* (IR: 0.91), and *An*. *calderoni* (IR: 1.75) were naturally infected with *Plasmodium* sp. (Table 3).

## 4. Discussion

The present study provides information on the composition, abundance, and natural infection of *Anopheles* specimens from northwestern and western Colombia. These areas encompass important endemic regions with high malaria transmission. The main vectors *An. nuneztovari*, *An. Albimanus,* and *An. darlingi* were the most abundant species in the localities sampled, which are in municipalities historically recognized for their high malaria incidence [29]. The fact that the main Colombian vectors are widely spread in these endemic regions suggest a high malaria risk for exposed humans and indicates a need for continued surveys to directed vector control strategies that are species-specific.

In northwestern Colombia, *An. albimanus* was the most abundant species and was widely distributed, detected in most localities. This is in congruence with previous studies that show high *An. albimanus* abundances in Colombian Atlantic coast localities [13]. Abundance of this species is mainly related to coastal localities, reflecting its ability to colonize salty water bodies [30,31]. In western Colombia, the most abundant species was *An. nuneztovari*; this is a finding that differs from previous reports for this region that show *An. albimanus* as the dominant species [13]. The difference in this study with respect to *An. nuneztovari* abundance in the west is related to the sample sites. Here, collections were performed more inland, in areas characterized by abundant forest land cover and mangroves [32], frequent in the Pacific region [33]. Furthermore, the sample sites are characterized by landscapes altered by human activities, such as open-pit mining [34]; these aspects correlate with the bionomy of *An. nuneztovari,* characterized by its ability to colonize artificial breeding sites [35] and its tolerance to anthropic environmental changes [32,36].

*Anopheles darlingi* was detected in similar abundances in both regions, western and northwestern Colombia. This species, considered the main vector in the Neotropics, in Colombia is distributed on both sides of the Andes [37]. Its occurrence in the northwest may be related to the presence of wetlands and rice cultivation areas detected in the sampled localities [36]. In the west, *An. darlingi* presence and relative abundance might be favored by the high rainfall that characterizes this region [38], which, altogether with the characteristic forest cover, propitiate the formation larval habitats without direct sunlight that favor the development of *An. darlingi* immature stages [10].

Furthermore, *An. triannulatus* was detected in both Colombian regions; it was the third most abundant species in the northwest, mostly because its predominance in two localities of the Sinú subregion. This species occurrence and abundance in these localities were previously reported [20,39,40], which indicates that in the localities persist the environmental favorable conditions for its presence. As such, in Colombia, *An. triannulatus* is mainly recognized as zoophilic [16,20], which may influence its abundance in localities where livestock farming is a common economic activity [12]. This species was scarce in the western region, only detected in one locality and in low number. Meanwhile, *An. braziliensis* was a species only collected in the northwest and restricted to localities of the Bajo Cauca subregion. This species was reported in the Zaragoza municipality almost 20 years ago [12]; however, the present study did not find *An. braziliensis* specimens in the localities surveyed of this municipality; this is possibly due to the landscape changes that have occurred in the region during in recent years [32]. Furthermore, this species was present in high abundance in the Nechí localities which had landscapes composed by bare soil, shrub cover, and wetland areas [36]. The differential distribution of species through time highlights the importance of carrying out continuous entomological surveillance to monitor species composition and transmission status.

This study detected twelve specimens naturally infected with *Plasmodium* spp.; *An. darlingi* was detected as being infected only in western Colombia, while *An. nuneztovari* and *An. albimanus* were detected infected in both regions. The infection rates detected for *An. nuneztovari* in the western localities (IR: 0.13–0.35) was similar to those previously reported in the northwest (IR: 0.1–0.04) [10,12]. Although *An. nuneztovari* infection rates were relatively low, the high densities detected and its preference for feeding on humans highlight its important role in transmission in these regions. Notably, this study presents the first records of *An. nuneztovari* natural infection in specimens from Istmina, in the west, and for *An. triannulatus* from Apartadó, in northwestern Colombia. In particular, *An*. *triannulatus* was previously reported to be naturally infected with *P. vivax* [10,20] and *P. falciparum* [17]; however, there is a lack of entomological or epidemiological evidence for its status [41] as a Colombian vector. In addition, this study is the first to report *An. braziliensis* from Colombia naturally infected with *Plasmodium* spp. This species has been reported to be naturally infected with *P. falciparum* and *P. vivax* in Brazil [42]; but it has not been incriminated regarding transmission in our country.

## 5. Conclusions

In this work, although species composition turnover was evidenced, the findings indicate that *An. darlingi*, *An. Albimanus,* and *An. nuneztovari* are the vectors primary responsible for maintaining malaria transmission in these endemic regions: northwestern and western Colombia. For species such as *An*. *triannulatus* and *An. braziliensis* detected to be naturally infected with *Plasmodium* spp., further studies on vectorial competence and sporozoite presence in salivary glands, along species survival, would help to elucidate their role as vectors in northwestern Colombia.

## Figures and Tables

**Table 1:** Composition and abundance of *Anopheles* mosquitoes collected in twenty municipalities of northwestern Colombia.

Species	Northwestern Region Colombia																				
	Antioquia Department												Córdoba Department								
	Bajo Cauca Subregion						Urabá Subregion						Sinú Subregion								
	ZAR	BAG	NECH	CAU	CAC	TAR	ARB	NEC	MUT	APT	TUR	SJU	MON	VAL	MOS	SAN	MOL	PTL	CAN	TAL	Total *n* (%)
*An. albimanus*	-	-	-	-	-	2	1368	77	8	17	118	27	14	16	278	527	36	4	247	5	2744 (27.8)
*An. nuneztovari*	56	557	2	22	83	4	13	21	64	56	51	-	21	821	4	-	43	710	-	107	2635 (26.7)
*An. triannulatus*	-	60	5	74	16	61	54	4	-	10	-	11	250	11	16	57	60	604	62	96	1451 (14.7)
*An. braziliensis*	-	1	840	118	3	32	2	-	-	-	-	-	-	-	-	-	-	-	-	-	996 (10.1)
*An. darlingi*	3	113	250	164	81	-	10	8	30	4	-	-	2	26	-	-	9	12	-	-	712 (7.2)
*An. punctimacula*	-	-	49	1	-	-	19	2	14	1	-	2	533	9	1	-	-	-	2	-	633 (6.4)
*An. albitarsis*	-	3	60	200	111	27	1	-	-	-	-	-	-	-	1	1	-	2	1	-	407 (4.1)
*An. pseudopunctipennis*	-	1	-	-	3	3	19	41	5	37	-	5	-	-	-	-	3	2	1	-	120 (1.2)
*An. neomaculipalpus* *	-	1	-	-	4	-	-	-	-	4	-	12	13	1	10	4	-	1	-	-	50 (0.5)
*An. oswaldoi*	-	-	-	20	1	-	-	-	-	-	-	-	-	-	-	-	7	14	-	-	42 (0.4)
*Near An. peryassui* *	-	-	39	-	-	-	-	-	-	-	-	-	-	-	-	-	-	-	-	-	39 (0.4)
*An. rangeli*	-	-	-	3	3	-	14	-	-	1	-	-	-	-	1	-	1	-	-	-	23 (0.2)
*An. apicimacula* *	-	-	-	-	1	1	-	-	-	1	-	7	-	-	-	-	-	-	-	-	10 (0.1)
*An. aquasalis*	-	-	-	-	-	-	-	-	-	-	-	-	-	-	-	4	-	-	-	-	4 (0.04)
*An. argyritarsis*	-	-	-	-	2	-	-	-	-	-	-	-	-	-	-	-	-	-	-	-	2 (0.02)
*An. malefactor* *	-	-	-	-	-	-	-	-	-	-	-	-	1	-	-	-	-	-	-	-	1 (0.01)
*An. calderoni* *	-	-	-	-	-	-	-	-	-	-	-	1	-	-	-	-	-	-	-	-	1 (0.01)
*An. squamifemur* **	-	-	-	-	1	-	-	-	-	-	-	-	-	-	-	-	-	-	-	-	1 (0.01)
Total	59	736	1245	602	309	130	1500	153	121	131	169	65	834	884	311	593	159	1349	313	208	9871 (100)

The three-letter acronyms correspond to the municipalities sampled in each region. ZAR: Zaragoza, BAG: El Bagre; NECH: Nechí; CAU: Caucasia; CAC: Cáceres; TAR: Tarazá; ARB: Arboletes; NEC: Necoclí; MUT: Mutatá; APT: Apartadó; TUR: Turbo; SJU: San Juan de Urabá; MON: Montería; VAL: Valencia; MOS: Moñitos; SAN: San Antero; MOL: Montelíbano; PTL: Puerto Libertador; CAN: Canalete; TAL: Tierralta. * The species was assigned by ITS2 and *COI* barcode [9]. ** The species was reported based on morphological identification.

**Table 2:** Composition and abundance of *Anopheles* mosquitoes collected in ten municipalities of western Colombia.

Species	Western Region Colombia										
	Chocó Department				Valle del Cauca Department				Nariño Department		
	Atrato-San Juan Subregions				Norte-Occidente-Sur Subregions				Costa Subregion		
	ATR	MAT	MSJ	IST	DAG	BUE	EZA	JAM	TUM	FPZ	Total *n* (%)
*An. nuneztovari*	86	3	570	289	-	779	-	-	-	-	1727 (51.6)
*An. darlingi*	152	487	70	270	-	-	-	-	-	-	979 (29.2)
*An. albimanus*	-	-	-	-	-	-	7	-	330	122	459 (13.7)
*An. calderoni*	1	-	-	-	1	-	30	8	55	57	152 (4.5)
*An. apicimacula*	3	-	10	-	-	-	-	-	1	-	14 (0.4)
*An. triannulatus*	-	9	-	-	-	-	-	-	-	-	9 (0.3)
*An. neivai*	-	-	1	-	-	-	-	-	-	2	3 (0.1)
*An. punctimacula*	-	1	-	1	-	-	-	-	-	-	2 (0.1)
*An. malefactor*	-	-	1	1	-	-	-	-	-	-	2 (0.1)
Total	242	500	652	561	1	779	37	8	386	181	3347 (100)

Acronyms of three-letters correspond to the names of the municipalities sampled. ATR: Atrato; MAT: Medio Atrato; MSJ: Medio San Juan; IST: Istmina; DAG: Dagua; BUE: Buenaventura; EZA: El Zarzal; JAM: Jamundí; TUM: Tumaco; FPZ: Francisco Pizarro.

**Table 3:** Detection of natural infection by *Plasmodium* spp. in *Anopheles* mosquitoes collected in northwestern and western Colombia.

Region	Municipality	Collection Date	Specimens Processed by ELISA (*n*)	Specimens Processed by PCR-Nested (*n*)	Infected Species (*n*)	IR, *Plasmodium* Species (CI)
Northwestern Colombia	ARB	2013 March	1490	469	*An. albimanus* ^b^ (1)	0.07, *Plasmodium* sp. (0.0000–0.004)
	APT	2014 March	131	120	*An. triannulatus* ^b^ (1)	10, *Plasmodium* sp. (0.002–0.552)
	CAU	2014 May	690	232	*An. braziliensis* ^b^ (1)	0.91, *Plasmodium* sp. (0.0002–0.0507)
	MOL	2014 July	159	113	*An. triannulatus* ^b^ (1)	1.67, *Plasmodium* sp. (0.0004–0.0929)
	PTL	2014 July	1349	461	*An. nuneztovari* ^b^ (1)	0.15, *Plasmodium* sp. (0.0000–0.0082)
	TAL	2014 October	496	164	*An. triannulatus* ^b^ (1)	0.35, *Plasmodium* sp. (0.0002–0.0398)
Western Colombia	ATR	2015 March	242	182	*An. darlingi* ^a,b^ (1)	0.66, *P. falciparum* (0.0002–0.0367)
	BUE	2015 May	779	270	*An. nuneztovari* ^b^ (1)	0.13, *Plasmodium* sp. (0.0000–0.0072)
	TUM	2015 August	345	181	*An. albimanus* ^a,b^ (1)	0.30, *P. falciparum* (0.0001–0.0169)
	FPZ	2015 August	170	121	*An. calderoni* ^b^ (1)	1.75, *Plasmodium* sp. (0.0004–0.0977).
	IST	2015 November	540	207	*An. nuneztovari* ^b,c^ (1) *An. darlingi* ^b,c^ (1)	0.35, *P. falciparum* (0.0001–0.0193) 0.37, *P. vivax* (0.0001–0.0206)

Specimens with detection of natural infection: ^a^ by ELISA. ^b^ by genus-specific nested PCR. ^c^ by species-specific nested PCR. IR%: infection rate. CI: confidence interval of the infection rate. All specimens detected naturally infected with *Plasmodium* were confirmed by PCR *COXIII*. Acronyms of three-letters correspond to the names of the municipalities sampled. ARB: Arboletes; APT: Apartadó; CAU: Caucasia; MOL: Montelíbano; PTL: Puerto Libertador; TAL: Tierralta; ATR: Atrato; BUE: Buenaventura; TUM: Tumaco; FPZ: Francisco Pizarro; IST: Istmina.

## Data Availability

All relevant data are presented within the manuscript.
